# What are participant beliefs regarding physical therapy led treatment? A qualitative study of people living with femoroacetabular impingement syndrome

**DOI:** 10.1016/j.bjpt.2024.101077

**Published:** 2024-05-21

**Authors:** Emily Bell, Andrea Mosler, Christian Barton, Denise Jones, Joshua Heerey, Richard Johnston, Sally Coburn, Joanne Kemp

**Affiliations:** a La Trobe Sport and Exercise Medicine Research Centre, School of Allied Health, Human Services and Sport, La Trobe University, Melbourne, Victoria, Australia; b La Trobe Sport and Exercise Medicine Research Centre and Discipline of Physiotherapy, School of Allied Health, Human Services and Sport, La Trobe University, Melbourne, Victoria, Australia; c Barwon Health, Geelong, Victoria, Australia

**Keywords:** Expectations, Hip pain, Interviews, Rehabilitation, Young adults

## Abstract

**Background:**

Physical therapist-led treatment programs are recommended for patients with femoroacetabular impingement (FAI) syndrome. Views of people with FAI syndrome regarding such interventions are currently unknown, including perceptions of potential barriers and facilitators to participation and adherence to exercise programs.

**Objectives:**

To explore participant perceptions of physical therapist-led programs for FAI syndrome, including barriers and facilitators for accessing physical therapy, and adhering to a rehabilitation program.

**Methods:**

Our qualitative study used semi-structured interviews to explore the perceptions of patients with FAI syndrome undertaking physical therapy-led treatment, where treatment was ceased due to Coronavirus Disease 2019 (COVID-19). The interview topic guide was informed by the Theoretical Domain Framework. Interviews were transcribed verbatim and data categories were developed using inductive thematic analysis. Themes were discussed between researchers until consensus was reached.

**Results:**

Fourteen participants (mean age: 30 years) with a range of physical activity backgrounds undertook interviews. We identified four key themes, 1) Patients believed their hip pain was caused by structural damage worsened through exercise; 2) Barriers and facilitators on the feasibility of physical therapist-led programs; 3) Participants held beliefs regarding the importance of adjunct treatments to exercise; and 4) Impact of FAI syndrome on physical activity participation.

**Conclusion:**

People with FAI syndrome believe they have structural damage which leads to their hip pain and are often afraid to exercise due to fear of causing more damage to their hip. Our findings suggest that people with FAI syndrome want clear education about exercise, imaging, and expectations of cost and duration of treatment.

## Highlights

• People with femoroacetabular impingement (FAI) syndrome believe they have structural damage which leads to their hip pain and are often afraid to exercise due to fear of causing more damage to their hip.
• People with FAI syndrome often highlight the costs as a potential barrier to engage in rehabilitation programs and want to know at the start of treatment how long treatment will last and what the cost will be.
• People with FAI syndrome are enthusiastic about using technology such as telehealth and exercise apps to help overcome some of the barriers in accessing regular face to face treatment.

## Introduction

Hip pain is a major cause of disability, significantly impacting quality of life, function, work capacity, and family life.^1^ It is commonly experienced by young to middle-aged adults (18–50 years), with the psychological and physical impact occurring at a time when major work and life responsibilities are experienced.^1^, ^2^, ^3^, ^4^ High economic burden from hip pain is currently experienced worldwide, with this projected to increase exponentially over the next three decades.^5^^,^^6^ Surgical intervention is common in this population,^7^ while quality of life and function remain low for these patients following surgery.^1^^,^^2^^,^^8^ Understanding differing classifications of hip pain might assist in the development of effective interventions that reduce the burden of this condition.

The International Hip Pain Research Network consensus meeting recommended that hip pain in young and middle-aged adults be classified as either femoroacetabular impingement (FAI) syndrome, acetabular dysplasia/hip instability, or other pathologies with normal hip morphology.^9^^,^^10^ Of these, FAI syndrome is the most common cause of hip pain in young adults, with hip osteoarthritis (OA) a potential sequalae. People with FAI syndrome also have pain, poor quality of life, physical impairments including reduced hip and trunk muscle strength, and reduced functional task performance.^9^^,^^11^^,^^12^ Treatment for FAI syndrome include surgical and non-surgical options, such as physical therapist-led interventions. In recent years, arthroscopic surgery has become an increasingly common treatment of FAI syndrome. However, this type of surgery is associated with significant cost,^13^ inherent risks,^14^ potential risk to cartilage health,^15^ and its longer-term efficacy is uncertain.^16^ A greater understanding of alternative treatments to surgery is needed.

The term “Physical therapist-led intervention” is broad, and may include aspects such as exercise therapy, education, manual therapy, and other therapies. These interventions may preserve the joint,^15^ and are recommended as an effective, low risk, and cost-saving alternative to surgery to reduce the symptoms of FAI syndrome in active individuals, although the ideal design of programs is unclear.^17^ A recent qualitative study determined that people with persistent hip pain tended to avoid physical activity as they believed their condition was exercise-induced.^18^ Recent consensus recommendations outlined physical therapist-led treatment as a critical part of management for FAI syndrome and consist of primarily exercise-therapy for at least 3 months duration,^17^ but can take up to 6 months.^19^ The onset of Coronavirus Disease 2019 (COVID-19) meant that physical therapy treatment ceased around the world, providing an opportunity to reflect on past practices, and explore new technologies that might enable contactless physical therapist-led treatment options for patients. Understanding the perspectives of the end-users is of utmost importance to ensure successful implementation. However, no studies have investigated patient understanding of physical therapist-led treatment, outcomes expected following participation, or their perceived barriers to participation.

The objectives of our study were to explore patient perceptions of physical therapist-led programs for FAI syndrome, including barriers and facilitators for accessing physical therapy; adhering to a rehabilitation program, and the impact of FAI syndrome on physical activity.

## Methods

### Study design

This qualitative study conducted semi-structured interviews with people taking part in the PhysioFIRST study for people with FAI syndrome.^19^ Ethics approval was obtained from La Trobe University Human Research Ethics Committee (#HEC17080) in compliance with the Helsinki declaration. The reporting of the study was undertaken according to the COnsolidated criteria for REporting Qualitative research (COREQ-32) criteria.^20^ Inception of this study was derived as a means of continuing to engage participants who had received minimal physical therapy intervention at the onset of the COVID-19 lockdown in Melbourne beginning in March 2020. This study did not alter the delivery of the intervention within the PhysioFIRST study, as the PhysioFIRST study was suspended while the current study was being conducted.

### Participants

All participants provided written informed consent prior to the study and were reminded of their rights prior to their interview. All participants were adults aged 18–50 years, males and females, recruited from the existing PhysioFIRST study cohort.^19^ Prior to the onset of COVID-19 restrictions, participants in this RCT had consented and just begun to undertake a physical therapist-led exercise program and education.

Full details of the exercise components of the two study arms are described in the published protocol paper as well as details of the cohort, recruitment procedures, and inclusion and exclusion criteria.^19^ A brief description is outlined below.

### Inclusion criteria

Details of inclusion into the physioFIRST study have been reported previously.^19^ Briefly, adults with i) activity-related hip and/or groin pain for more than 6 weeks, ii) a positive Flexion-Adduction-Internal Rotation (FADIR) test,^9^ and iii) indication of cam morphology, defined by an alpha angle greater than 60° on a supine anteroposterior pelvic or Dunn 45° radiograph were considered eligible.^9^^,^^21^

### Recruitment procedures

The initial COVID-19 lockdown occurred in Melbourne, Victoria between March and June 2020. Government regulated closure of gymnasiums and restriction of access to allied health services significantly impacted the PhysioFIRST trial participants. All participants in this study were individuals whose participation in the PhysioFIRST study was suspended due to these restrictions, whereby participation aimed to maintain engagement of participants during the lockdown and improve delivery of future physical therapist-led programs. They were receiving no active physical therapy at the time of the study. They were contacted by email in April 2020 and invited to participate in this qualitative study. Interviews were conducted in May 2020 via the Zoom platform during the COVID-19 lockdown.

### Data collection

The topic guide (Supplementary material) for the interview questions was developed by four authors (EB, AM, CB, JK) based on the Theoretical Domain Framework^22^ and included questions relating to knowledge, skills, identity, beliefs, and goals. Interview schedules were developed in consultation with an experienced qualitative researcher (CB) and experienced FAI syndrome clinicians and researchers (JK and AM). The authors have a range of 4–28 years of clinical experience treating patients with hip pain. Semi-structured interviews allowed us to explore key themes related to people with FAI syndrome and allowed flexibility for participants to discuss what they felt was important to them in relation to FAI syndrome.

Demographic data including age, sex, body mass index (BMI), duration of symptoms (months) and International Hip Outcome Tool (iHOT-33)^23^^,^^24^ were collected to characterise the cohort. The iHOT-33 is a recommended measure of hip-related quality of life in young adults with hip pain and is scored from 0 to 100, where 100 is the best possible score.^23^^,^^24^

Interviews utilised open-ended questions and were conducted via Zoom by a female physical therapist (EB) with previous qualitative study experience^25^^,^^26^ and no prior relationship with any study participants. Interviews were audio recorded, no repeat interviews were conducted, and field notes were not collected. To facilitate ongoing engagement with the physioFIRST trial at the onset of the COVID-19 lockdown in Melbourne, Australia, we included all eligible participants who consented.

### Data analysis

From an epistemological perspective, the approach used during the interviews was interpretive/constructive, because the study aimed to gather information from the perspective of the interviewed participants. The interview questions were open ended with no right or wrong answer but provided a framework for the descriptive process. Interviews were transcribed verbatim by an experienced research assistant at La Trobe University and imported into the NVivo qualitative data analysis software (QSR International Pty Ltd, Melbourne, Australia, Version 12, 2020). Participants were provided with their interview transcript immediately following transcription and given the opportunity to elaborate on statements made in their interview. No participants made amendments. Qualitative analysis of the interview data commenced with a close review of each transcript by one researcher (EB) to gain familiarisation with the dataset. Relativism was the ontological philosophical approach which guided the coding such that the responses of the participants created the constructs and developed the themes for the analysis.

Data analysis followed reflexive thematic analysis,^27^ which included i) a code being assigned to each key issue, ii) similar codes were grouped and findings were identified as subthemes, and iii) subthemes were grouped to form overarching themes. All transcripts contributed to the final themes identified, themes were not predetermined. As data saturation is not aligned with reflexive thematic analysis,^28^ we did not seek data saturation. To ensure data accuracy, a random sample of 50 % of transcripts were also coded independently by a second researcher (AM). Generation of initial themes took place between researchers (EB, AM, CB, JK). Themes evolved during four online meetings every two weeks, to ensure a deep, nuanced understanding of the data.

## Results

**Fig. 1:**
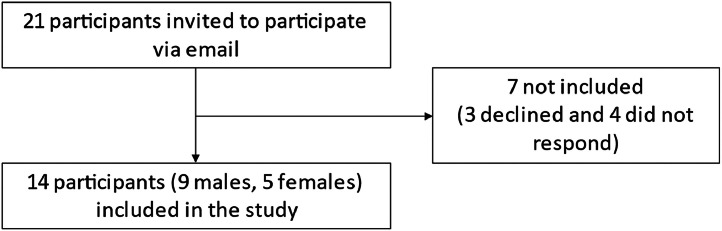
Flowchart of participant recruitment.

Twenty-one participants were invited to participate in this study via email. Fourteen participants responded to the invitation and agreed to take part in the study, three declined and four did not respond (Fig. 1). Interviews were conducted and analysed for all 14 respondents (9 males and 5 females). Interviews lasted an average of 19 min (range 12–31). Participants interviewed had a mean age of (standard deviation) 30 (10) years of age, BMI of 23.2 (3.5) kg/m^2^, and 51 (43) months of symptoms. Participants had been undertaking the PhysioFIRST exercise protocol for a mean of two weeks (2), and the mean baseline iHOT-33 score for this cohort was 55.4 (18.9).

### Summary of themes

Four themes were identified from interviews. 1) Patients believed that their hip pain was caused by structural damage and worsened through exercise (Table 1). 2) Barriers and facilitators on the feasibility of physical therapist-led programs (Table 2); 3) Participants held beliefs regarding the importance of adjunct treatments to exercise (Table 3); and 4) Impact of FAI syndrome on physical activity participation (Table 4). Within each theme a number of subthemes emerged, providing a more detailed and nuanced understanding of participants’ perceptions of physical therapist-led programs for FAI syndrome, barriers and facilitators that exist for accessing physical therapy, and challenges and successes for adhering to a rehabilitation program.

**Table 1:** Patients believed their hip pain was caused by structural damage and worsened through exercise.

*Subtheme:*	*Findings:*	*Illustrative quotes:*
Participants hold biomechanical beliefs about the impact of exercise and symptoms on their hip	Some participants believe exercise is a cause and an aggravating factor for their hip pain; others perceive lack of exercise contributes to increased symptoms.	“I think my exercise flares it up.” – P1 “I think running is quite painful. And I push through it because I can, but I get frustrated by the pain in the hip particularly afterwards.” – P1 “Possibly lack of strength or just general exercise around it might aggravate it as well.” – P11
	Participants describe the underlying morphology and intra-articular pathology, and its interaction with activity as the cause of their hip pain.	“I guess the actual condition itself; I can't remember the name of it, but the physical shape of my hip bone and the way it interacts with the socket and from what I can gather there's not that much information of what causes that.” – P11
	Some participants believe that long standing lower limb musculoskeletal issues are also the cause of their hip pain.	“I guess the longest-standing pain I have is in the hamstrings. I suppose that tightness in my hamstrings might have put my hips out of joint and brought some discomfort to the front of my hip.” – P5

**Table 2:** Barriers and facilitators on the feasibility of physical therapist-led programs.

*Subtheme:*	*Findings:*	*Illustrative quotes:*
Perceptions of a reasonable commitment to gain benefit vary (and are influenced by several factors).	There is variability in participants’ expectations of contact frequency with physical therapists, which is dictated by their pain and functional limitations.	“I guess it depends on the pain, but at the moment every couple of weeks if necessary…3 months.” – P5 “I would imagine maybe to try solve the initial issue, maybe once a fortnight for maybe two or three sessions of that. And then hopefully drop it back to once a month. Just for fine tuning, or check-ups.” – P1
	Considerable variability is reported by participants regarding frequency and duration of physical therapy treatments.	“I would appreciate being able to go every couple of weeks…Half an hour.” – P5 “Oh certainly once a week… I guess with my perception, since it's been a constant issue for so many years, I wouldn't imagine it's going to be a quick fix, so I'm not that sort of silly about it all. I would expect for me personally is something around a six-month therapy process. That would be a lot more realistic.” – P7
Home exercise programs.	Participants provided a range of what they considered to be an acceptable commitment to home exercises.	“…3 times a week would be a reasonable commitment.” – P4 “I'm pretty happy doing a daily workout.” – P5
	Participants acknowledged that the length of home exercise sessions would vary depending on their program	“…maybe 20 to 30 min for the exercises.” – P9 “30 min to an hour, depending on what they are I guess.” – P8
	Participants who already exercise frequently acknowledged a hip-specific home exercise program would be in addition to their usual activities or sport.	“And maybe 3 to 4 days per week exercise program. Obviously I have to factor in that I exercise most days a week anyway, and it's a lot more strenuous than maybe a physio exercise program would be. So I'd have to incorporate that into my regular routine. So maybe 3 to 4 days a week.” – P9
What participants want from education as part of physical therapist-led programs.	Participants want a clear understanding of the problem and cause of their hip pain.	“Well I would hope I would have a better understanding about what is actually causing it. I think that's an important part of how we get better from these sort of things.” – P9 “I guess just having an understanding of what the issue is, and I guess why it occurs and what prevents it from occurring in a normal, functional hip. And why my hip might not necessarily be functional.” – P12
	Participants gained benefit from having imaging clearly explained to them.	“I really loved when physio X actually showed me what I was looking at in the MRI, cause I'd never actually seen any of my x-rays or MRIs before. Her pointing and showing to me… Actually seeing it and understanding it - cause I'm a very visual learner…” – P10
	Education is supported by realistic goal setting. Participants like to have expectations about long term management and prognosis set out from early in physical therapy interaction and intervention.	“The actual prospects of improvement…Yeah I guess the more information you've got, the better.” – P11 “Where I'm at, the progress physically that I'm making, whether it's increasing, decreasing from a physical point-of-view… Otherwise you're just sort of feeling like you're working in the dark almost.” – P7
	Participant education needs to be tailored to individuals learning style and knowledge gaps.	“I'm hoping I can do what I need to do without fear of reinjuring it. That's really my fear… But the pain's still there. So I hope going out for a run, or going to footy training or whatever, doesn't result in me having that concern, that anxiety over my hip. And I know that it's structurally sound and I can do what I need to do on it.” – P9
	Health professionals can instil both positive and negative beliefs influencing exercise participation	“I was sort of lead to believe [by my health professional] I had torn my hip flexor… exertion or running or other exercise was sort of just me re-tearing that structure. But it's persisted for quite a while… I guess I don't have too much of a background of an understanding of what caused it… So yeah I guess I've just sort of come to that functional understanding of what I need to do without knowing what exactly is the cause behind it.” – P9
Barriers to treatment access	Competing commitments including work, university, and childcare are a barrier to accessing physical therapy for many people with FAI syndrome.	“Time- I work full-time plus I obviously got the family, so you really have to manage your time well to slot that into your week…” – P11 “Well I'm a pretty busy person, I work three days a week, I do research as well. I had extra-curricular activities too and obviously a family…” – P9
	Participants express that travel requirements to attend face-to-face appointments regularly are a barrier for seeking support from physical therapists.	“A barrier is distance to the physio…” – P2 “I think just for me travel is a little bit out the way.” – P14
	Participants acknowledge that there is a cost associated with physical therapy or upkeep of a rehabilitation program for their FAI syndrome. Some participants report having limited access to physical therapy due to costs associated with treatment.	“Yeah okay so cost is obviously one, especially if you had to go weekly. And that's not unusual to go weekly to physio in my experience.” -P4 “I probably reckon I've spent $2000 in the last two years. So yeah you know, $1500 a year probably is a reasonable number. Maybe including a gym membership, probably $2500.” – P8
	Comorbidities may impact participants adherence to physical therapy programs.	“Yeah so other injuries is definitely something that's inhibited me the last few weeks…” -P5 “…my normal regular health, so with my low blood pressure and thyroid and iron, I'm light-headed all the time. Like I don't do well in hot weather…” – P10
	Motivation can act as a barrier to adherence to home exercise programs.	“And then doing exercises at home, I guess it's just sort of… not so much a time constraint thing, but more a motivational constraint thing. Being at home its sort of hard to do a lot especially after a workout with exercise and stuff, just that extra bit.” – P14
	Access to workout space and equipment may be a barrier to some people with FAI syndrome progressing home exercise programs.	“Probably access to the gym… would be a barrier as well.” – P4
	Participants’ expectations varied, however they acknowledge that the cost of physical therapy is set per session and the total cost to manage an issue is dictated by how many visits to the physical therapist are required.	“I think that would have been very expensive. So that would have not been an option for me, I would have just been like stretch, keep the muscle warm sort-of thing, but I know the industry is expensive and it would have been a couple of hundred a session.” – P3 “I don't have much idea of what like physio sessions and that cost, but maybe like $50?.. I'd probably go once-a-month if I'm lucky then.” – P13
Facilitators to treatment access.	Participants report that the potential to improve their hip pain acts as motivation to prioritise access and adhering to physical therapy.	“…And I have to stay motivated. So, I have to feel like I'm getting some incremental rewards by… you know, less hip pain or to be able to keep doing the things that I'm doing. Then I feel that's the reward I need to hopefully stay motivated and keep on the program” – P1
	Planning treatment sessions and tailoring home exercise routines around the participant's lifestyle and needs are perceived as an enabler to physical therapy.	“Just having a clear plan… set physio dates, set a regime that you can stick to that you can lay out 3-months in advance. As long as you know what's happening, you can sort of tailor your life around that usually… So I guess having a good plan, and some sort of flexibility as well as you move along.” – P11
	Subsidised access to physical therapy or equipment acts as a facilitator to physical therapy and exercise adherence.	“Sure, so financial incentive I guess, having the study support me to see a physio has been really beneficial for me. And it's giving me a kick up the pants, so-to-speak, about reducing that hurdle to be able get into the routine and having support to be able motivate myself. So that's been a real big benefit.” – P5 “…if you're paying $50-$60 for a physio appointment regularly, it's probably fair enough that they provide some TheraBand's for you, I think that's nice of them. You're probably likely to get adherence that way.” – P4
Using technology to enhance treatment.	Alternate forms of communication with a physical therapist such as video telehealth or a messaging service are seen as favourable by participants to save travel time and expense.	“I guess if you're doing it over video, you can probably cut out a bit of the costs cause you're not making the trip there.” – P14 “Maybe just like being able to message my physio or whatever and say 'I'm up to this set of exercises' or whatever or 'I'm doing this one and it's really painful' or 'I'm finding it really easy, what one should I do instead' or 'What should I do?'. Instead of having to go to them all the time.” -P13
	Participants reported having the option of face to face or telehealth would suit them and their circumstances at the time.	“A Zoom session would potentially help and be less of a barrier.” – P2 “I think it's actually a blessing in disguise being able to do physio from home if that's still going to be an option going forward…. just checking in with the physio every now-and-then and being able to do that via video rather than having to go in person. That could be a really big… I guess it would help me with time commitment. And yeah it would be pretty useful.” – P12
Facilitators to rehabilitation programs.	Most participants state that routine and accountability are enablers to adhering to a rehabilitation program.	“I actually also think the reverse, going to the physio would make me do it more as well. Cause it sort of makes me accountable to check in….I guess just setting myself more structured program or goals, like what time of the day to do it.” – P14 “A text or reminder, or just personally putting blocking out a space in the calendar to do it.” – P6
	Another enabler to adherence included seeing progress related to their home exercise program.	“I'm actually going up the levels, and actually seeing and being told on a piece of paper, 'Oh like you were here a couple of weeks ago, but now you're up to here'…” – P10 “Just seeing results.” – P7
Expected outcomes and goals provide motivation within physical therapist-led programs	Participants expect hip pain to be reduced and manageable with physical therapy intervention.	“Movement, freedom to be able sit and stand for long periods without pain or without it taking mental bandwidth that there's some uncomfortableness there.” – P2 “I would hope there would be a reduction in pain. And to keep it mobile. Just to keep it in you know, whatever condition so that I can keep using it as long as I can.” – P1
	Participants expect to be able to return to sport and activities that have previously been restricted due to hip pain.	“Probably an increase in the ability to complete as much physical activity as I want. And to feel confident that it's not going to flare up I suppose, often.” – P4 “I play netball, I've just gone back to playing netball … And it was alright, but I'd like to run maybe 5 km comfortably.” - P13
	Participants want to avoid or prolong the need for hip surgery.	“…hopefully, long-term, reduce the risk of doing long-term damage to the hip, and ending up needing a hip replacement… But if there is any risk of that happening, I'm hoping to get the tools to be able to avoid that going forward.” – P12
	Participants describe their desire to return to usual activities including engagement with family and sport because of physical therapy for their FAI syndrome.	“Well just to get back my normal performance, I was running quite a bit and before I was running every second day also. I enjoy running, and I was running about half a marathon every weekend.” – P3 “I hope increased flexibility, associated mobility increases, and reduced pain.” – P5

**Table 3:** Participants held beliefs regarding the importance of adjunct treatments to exercise.

*Subtheme:*	*Findings:*	*Illustrative quotes:*
Use of medication has positive and negative associations	Some participants use over the counter medication to manage spikes in hip pain. Medications reported included paracetamol and non-steroidal anti-inflammatory drugs.	“Oh I take Panadol sometimes, when I wake up and I'm really stiff and sore… sometimes the pain just pisses me off and I have to deal with the kids and I'm not in the mood so I'll just take a Panadol.” – P1 “Maybe occasionally a Voltaren or two when it was really bad, just to decrease the inflammation but that's it.” – P9
	Several participants report not using any medication for their hip pain.	“Even Panadol, Nurofen, everyone takes it and they feel better and for me I just… I don't know, it doesn't take it away… just something to even take the edge off it. I would love something that could just do that. But then I don't want to be on medication the rest of my life, I want this to be– I only want it to be a temporary thing.” – P10 “Nah that's all rubbish that stuff, it doesn't work…” – P2
Adjunct therapies provided by health professional	Some participants have seen physical therapists for their FAI syndrome previously which included different variations of manual therapy and exercise-therapy and perceived differences in their current experiences.	“Just do the dry needling, that was a while ago… Um manual therapy… And just a bit of gentle stretching.” – P1 “Yeah I've seen the physio– like two different physios for it. They were both predominantly exercises for it. One of the physios was strengthening the adductors, and the other one was doing more the glute sort of stuff. Obviously both of them didn't really do anything. But yeah now this is a different approach again.” – P14
	Some participants have seen health professionals other than physical therapists.	“Well the myo is more massage, and that was more when I had… the problems were less… it was just my core and hip sort of, as opposed to everything else. And so just massage on the hip and quad really…I did dry needling with the myotherapist… Yeah helpful I think. It feels funny.” – P8 “The osteo was going more with like… groin stretches and… opening up rather than closing up… That sort of resistance sort of method that they use like where you push against them and release, push against them and release.” – P11

**Table 4:** Impact of FAI syndrome on physical activity participation.

*Subtheme:*	*Findings:*	*Illustrative quotes:*
Usual physical activity levels of participants	There is considerable variation in participants’ physical activity levels at point of entry into the program	“So last week I think we ran about 32 kms last week and I had quite a lot of pain… So that would be my normal, 30–40 kms would be my normal.” – P1 “I don't really exercise a lot… I guess I'm on my feet all day and I'm walking the whole time… So that's probably the extent of it. And then weight-lifting children. Yeah I've been pretty slack exercise-wise in the more recent part of my life.” – P11 “Probably training for footy twice a week, and then I guess just doing weights training maybe 3 times a week.” – P12
FAI syndrome has a variable impact on physical activity	Pain impacts physical activity participation for most participants.	“I feel like it has this week, because I pushed it into the red zone a bit, being stubborn. And I guess as you can see, because I'm not doing the other stuff for reasons I can't explain, then I do more of the running and then that messes it up. So yes, it has impacted this week, by me only doing it last week. So lessening activity due to pain, yep.” – P1 “Not always, but yeah like I said I do get flare-ups occasionally and when it does it will impact on my ability to do everything that I want. Often it's just a case of rest until the pain subsides enough to be able to continue the activities. It varies. Sometimes it might be 2 to 3 days, up to… it could be a week and a bit.” – P12
	Pain does not impact physical activity for some participants.	“Not really, it doesn't really affect me when I am being active. Itʼs more when Iʼm stationary that yeah… When I'm active, itʼs not painful. But being stationary is probably the biggest issue for me.” – P11
Patient perceptions of optimal physical activity / prescribed exercise for their hip (e.g. preferences, safety, dose)	Participants describe the need to modulate prescribed exercise and self-direct activity to feel their best.	“And if it's hurting, then take back a step from exercise. Or if I'm doing the treatment and it's too much for me, I'll just do two reps instead of four. Just take it down a notch.” – P10 “I'd probably say 2 h a day of at least just moving around. 2 to 3 h a day. Maybe a bit more? 4?” – P11
	Participants describe their perception of the physical activity guidelines.	“It can be pretty invasive. An hour of intense exercise, I donʼt think anyone gets that unless you're an elite athlete… Sure. I mean, itʼs very vague isn't it. You can say I can have half an hour of moderate exercise, or I can have an hour of vigorous exercise, like thereʼs a big gap between those two. I don't know what that means. It becomes so watered down, it doesnʼt dictate what you should be doing…” – P5
	Participants describe their perception of undertraining.	“Too little exercise is bad. Very bad. At least 10 min every couple of days of stretching it out. Minimum. Definitely.” – P7 “…And then too less, yeah definitely. Probably 3 or 4 days in a row just sitting still not doing exercise, that's when it feels really bad…” – P2
	Participants discuss their perceptions of prescribed exercise required for their hip.	“Oh probably an hour a day. 6… You need a day off. Yeah you can certainly do too much, and too much is probably doing squats and four or five leg actions - that's kind of too much.” – P3 “Yeah I think like I said probably 30 min of those specific hip exercises 3 times per week. Maybe 3 or 4 times but I think that's probably it. And then add on if I were going to the gym and having other strengthening sessions that would contribute towards it, I think that would probably be optimal. But at the moment, not possible… - P4
Barriers to physical activity.	Current circumstances may act as a barrier to physical activity.	“If I canʼt sleep well, then I probably wonʼt do it the next day if I haven't exercised, just cause I physically can't.” – P10 “…So just depending what work was like or my family situation, I'd get it done either once or twice a morning a week.” – P10
Facilitators to physical activity	Participants described various facilitators that can enhance their physical activity levels.	“And maybe diarising… having some sort of mental plan”- P1 “…feedback of your pain scale. So if I did two 10 km runs last week, and then could barely walk two days after, maybe that reminds me that's pushing too much.” – P1

#### Theme 1: patients believed hip pain was caused by structural damage and worsened through exercise (Table 1)

An association between undertaking exercise and provoking symptoms was identified, with some participants suggesting that a lack of exercise had the potential to exacerbate symptoms. In contrast, strong biomechanical beliefs about the impact of exercise on hip pain were voiced, with other participants believing that exercise may damage their hip and worsen symptoms (Table 1). Influence from health professionals may lead to positive or negative beliefs about the safety and benefits of exercise. Interpretation of imaging findings further enhanced belief in the ‘cause and effect’ biomechanical model.

#### Theme 2: barriers and facilitators on the feasibility of physical therapist-led programs (Table 2)

Variability in perceptions of a feasible and reasonable commitment to a physical therapy-led program was a key finding, influenced by several factors. Commonly, participants thought physical therapist-led programs should include more frequent physical therapist contact during initial rehabilitation. Expectations for the duration of physical therapy sessions were influenced by participants prior experience and were less than one hour. Greater differences were evident in relation to home exercise programs, potentially reflective of the range of physical activity undertaken by participants outside the parameters of the physical therapist-led program. An acceptable duration for home exercise sessions ranged between 20 min and 1 h, with a handful of participants suggesting that combining their rehabilitation exercises with their usual routine may facilitate adherence.

Education provided by a physical therapist contributed to participant's expectations of management and prognosis and provided reassurance. Participants suggested that appealing to different learning types (e.g. audio, visual, tactile) would facilitate understanding. Barriers to physical therapist-led programs included opportunity (competing commitments), perceived lack of time, access (requirement for travel, access to equipment/space), cost, and motivation. While facilitators included use of technology assisting to address opportunity and access barriers, improvements seen from participation in physical therapist-led programs, motivation to prolong or prevent the need for surgical intervention, and motivation to return to usual activities.

Diversity in the delivery of components of a physical therapy-led program were valued, with technology and telehealth enhancing opportunities for education, motivation, reassurance, and minimisation of cost. Expected outcomes of physical therapist-led programs for FAI syndrome included reduced pain and symptoms, the ability to return to activities of daily living and sport (if applicable), as well as the potential to delay or eliminate the need for surgical intervention. The likelihood of meeting expectations and joint goal setting impacts feasibility of physical therapist-led programs as people with FAI are more likely to engage in programs that meet their expectations. Facilitators for treatment access included motivational factors such as ability to reduce hip pain. Subsidised cost of physical therapy and access to equipment increased the likelihood of participating in physical therapy treatment, and increased exercise adherence. Facilitators to rehabilitation programs included accountability through supervised exercise and seeing progress towards their goals. Routine was perceived as a facilitator to rehabilitation programs and physical activity participation.

Having the choice of face-to-face treatment or telehealth was perceived as a facilitator to accessing physical therapy, allowing participants to choose what catered best to their individual needs.

#### Theme 3: participants held beliefs regarding the importance of adjunct treatments to exercise (Table 3)

Participants engaged in additional treatments including; medications and adjunct therapies. Perceived effect of over-the-counter medications such as paracetamol and ibuprofen varied. Some participants described self-dosing as they felt it was necessary, while others described choosing not to use them with the belief that they don't help their hip pain.

Adjunct therapies participants described having tried included dry needling, massage therapy, and stretching. No participants mentioned previous visits to general practitioners or orthopaedic surgeons when asked if they had seen other health professionals for their FAI syndrome.

#### Theme 4: impact of FAI syndrome on physical activity participation (Table 4)

Participants reported dramatically contrasting levels of usual physical activity, ranging from minimal activity aside from occupational activity; to running more than 30 kms per week. Perceived optimal physical activity levels depended on the individual participant's lifestyles and pain levels. Some participants described the need to gradually build the strength and exercise tolerance with graded programs. Participants cited perceived risks of over- or under-exercising, with varied perceptions of what was an appropriate level of physical activity or exercise. Barriers to physical activity included individual circumstances such as work commitments or poor sleep. Facilitators included routine, accountability (e.g. diary), and pain tracking.

## Discussion

We explored the beliefs of people with FAI syndrome who had just commenced a physical therapist-led treatment program. Specifically, we sought to understand patient perceptions of physical therapist-led treatment for FAI syndrome, including barriers and facilitators for accessing physical therapy, adhering to a rehabilitation program, and the impact of FAI syndrome on physical activity. Interestingly, our findings are similar to qualitative studies of people with shoulder, low back, and knee pain, which imply that clinicians should be aware of the large psychosocial impact of musculoskeletal pain and the positive and negative influences that clinicians can have, supporting the need for patient-informed treatments for FAI syndrome.^29^, ^30^, ^31^

All participants held strongly to a biomedical model of pain, believing that their hip pain was related to bony morphology and intra-articular soft tissue pathology reported in imaging, and that exercise would damage already-damaged hip joint structures further. These beliefs are consistent with an older cohort of people with hip pain who had been referred to an orthopaedic surgeon.^18^ The expressed belief in the biomedical model of pain is likely to have influenced the barriers to exercise identified in Themes 2 and 4, suggesting an intersection of these themes, and a strong need for education in these patients. Substantial evidence indicates that the relationship of hip pain with hip joint morphology and pathology is poor. A recent systematic review indicates the prevalence of labral tears in symptomatic individuals is higher (62 %; 95 % CI: 47 %, 75 %), and similar to those without symptoms (54 %; 95 % CI: 41 %, 66 %).^32^ Additionally, while cam morphology typically seen in FAI syndrome appears to be more common in people with symptoms compared to those without (49% vs 23 %),^33^ prevalence in athletes is high at approximately two-thirds regardless of symptoms.^34^, ^35^, ^36^ People with FAI syndrome should be informed of the uncertain relationship between hip pain and structure, and be given the opportunity to discuss this relationship with their treating clinician.

Interestingly, our participants’ identified that they expect pain to be reduced following exercise intervention (Theme 2) but also that pain was a barrier to performing physical activity (Theme 4) Contrary to popular belief that exercise worsens hip pain, exercise-based treatment is recommended as a key intervention for young and middle-aged adults with FAI syndrome.^17^ Current evidence demonstrates that exercise-therapy improves pain and function with moderate effect,^16^ and may preserve the joint.^15^^,^^16^ Our findings suggest that the sports medicine community needs to work hard to change commonly held negative beliefs about the relationship between exercise and joint health in people with FAI syndrome. Education elements (e.g. audio, visual, tactile) within physical therapist-led programs for FAI syndrome need to inform patients of the benefits of exercise-therapy for pain and function, and that activity such as running may actually protect the joint against deterioration in joint structure, as recently reported in relation to knee joint health.^37^^,^^38^ Physical therapists should also consider exploring with patients why they maintain beliefs about the danger of exercise, and ensure that education is tailored to address individual patient's beliefs.

Several non-surgical treatment options were perceived as helpful in conjunction with exercise. Participants described a willingness to use over the counter medication to reduce pain and facilitate greater physical activity participation. In people with hip OA, non-steroidal anti-inflammatory medications have greater benefits than paracetamol, but also have a higher risk profile.^39^ Clinicians should consider encouraging patients to consult their general practitioner about safe and effective use of over-the-counter medication. Several participants reported seeing clinicians for other therapies such as massage, dry needling, and stretching. Patients perceived varied levels of benefit from adjunct therapies. To date, there is no evidence whether these adjunct therapies provide additional benefit over exercise-therapy for patients with FAI syndrome.^17^ Interestingly, a recent study of people with hip OA found that while both exercise therapy and manual therapy were cost effective compared to usual care provided by a general practitioner, the cost effectiveness was superior in the exercise therapy group and only exercise therapy proved to be clinically effective.^40^ While these findings have not yet been confirmed in younger people with FAI syndrome, they suggest that clinicians should consider treatments such as manual therapy as adjuncts only, and should not be prioritised over exercise-based treatments.^17^

Although the participants in this study represented a wide range of activity involvement, some demonstrated a hesitancy to undertake physical activity due to fear of creating more damage, while others were more confident to push into pain. This finding supports recently published work where an older group of patients (mean age 51 years) with hip pain who had been referred to an orthopaedic surgeon were also afraid of exercise and its effect on joint damage.^18^ The World Health Organisation's 2020 guidelines for physical activity stated that all adults should undertake 150–300 min of moderate-intensity, or 75–150 min of vigorous-intensity physical activity each week, as well as regular muscle strengthening activities.^41^ It is imperative that physical therapists ensure that patients with FAI syndrome are working towards this goal.^42^ Reassurance and education is essential^17^ to guide patients that moderate amounts of targeted and guided physical activity and exercise do not cause joint damage, instead they may help maintain a healthy joint as tissues respond to load.^15^^,^^37^^,^^43^ Physical therapists should also adapt exercise programs to suit the individual patient's preferences, which may include a graded approach to the resumption of activity to help overcome patients’ fears.^19^

In summary, tips for physiotherapists to use when designing rehabilitation programs for patients with FAI syndrome include (i) providing clear education about the benefits of exercise and physical activity, and the poor relationship between structure and symptoms; (ii) inform patients of the costs and duration of treatment required, and the commitment to exercise required (2–3 times/week for at least 3 months); (iii) embrace technologies such as exercise apps and telehealth appointments; (iv) make sure patients have access to equipment needed for rehabilitation; (v) make sure return to sport and physical activities are prioritised; (vi) consider using adjunctive therapies and referring for simple analgesia

### Strengths and limitations

Our study has several strengths and limitations that should be highlighted. Our study includes a somewhat diverse population of people with FAI syndrome, including a large variation in symptom duration which may have contributed to varied perspectives and experiences. We included 14 participants which might be considered a smallish sample for qualitative research. We used Zoom to conduct and record the qualitative interviews. It is possible that participants may have provided different responses if the interviews were conducted via telephone call or in person. We used a Theoretical Domain Framework approach and reported our findings according to the COREQ-32 criteria. The population we recruited responded to advertising to participate in a clinical trial of physical therapy for FAI syndrome, meaning that they might have preferred not to undergo surgery, and may be more enthusiastic towards physical therapist-led treatment and exercise therapy. Our cohort was more likely to perceive prolonged physical therapist-led treatment as acceptable compared to people who are seeking a fast solution. Lastly, and possibly most importantly, we only included white patients from a large metropolitan area in a high-income country. The findings of our study and recommendations made may not be appropriate or possible for people living with FAI syndrome who are less well-resourced. A recent systematic review showed that physical therapy is most likely to be accessed by people who are white, well-educated, live in an urban environment, have access to transport, are employed, have high socioeconomic status, and are privately insured.^44^

## Conclusion

Our findings indicate that people with FAI syndrome who had just commenced a physical therapist-led treatment program believe hip pain is caused by structural features and that they are often afraid to exercise due to fear of causing more damage to their hip. Not all patients have the resources to be able to undertake physical therapist-led treatment. Our study findings reveal important insights about how to facilitate successful implementation of best-practice physical therapist-led treatment for people with FAI syndrome. We recommend physical therapists provide clear education about the benefits of exercise and the poor relationship between structure and symptoms, inform patients of the costs and duration of treatment required, and embrace technologies such as exercise apps and telehealth appointments.

## Conflicts of interest

The authors have no conflicts of interest to declare

## Appendix Supplementary materials

Image, application 1

## References

[1] Filbay S.R., Kemp J.L., Ackerman I.N., Crossley K.M. (2016). Quality of life impairments after hip arthroscopy in people with hip chondropathy. J Hip Preserv Surg.

[2] Kemp J.L., Makdissi M., Schache A.G., Finch C.F., Pritchard M.G., Crossley K.M. (2016). Is quality of life following hip arthroscopy in patients with chondrolabral pathology associated with impairments in hip strength or range of motion?. Knee Surg, Sports Traumatol, Arthrosc.

[3] Ackerman I.N., Kemp J.L., Crossley K.M., Culvenor A.G., Hinman R.S. (2017). Hip and knee osteoarthritis affects younger people, too. J Orthop Sports Phys Therapy.

[4] Ackerman I.N., Ademi Z., Osborne R.H., Liew D. (2013). Comparison of health-related quality of life, work status, and health care utilization and costs according to hip and knee joint disease severity: a national Australian study. Phys Ther.

[5] Cross M., Smith E., Hoy D. (2014). The global burden of hip and knee osteoarthritis: estimates from the global burden of disease 2010 study. Ann Rheum Dis.

[6] Ackerman I.N., Bohensky M.A., Zomer E. (2019). The projected burden of primary total knee and hip replacement for osteoarthritis in Australia to the year 2030. BMC Musculoskelet Disord.

[7] Coburn S.L., Ackerman I.N., Bohensky M.A. (2020). What are the rates of hip arthroscopy in Victoria, Australia and Denmark from 2012 to 2018. Osteoarthritis Cartilage.

[8] Ishøi L., Thorborg K., Kraemer O., Hölmich P. (2018). Return to sport and performance after hip arthroscopy for femoroacetabular impingement in 18- to 30-year-old athletes: a cross-sectional cohort study of 189 athletes. Am J Sports Med.

[9] Reiman M.P., Agricola R., Kemp J.L. (2020). Consensus recommendations on the classification, definition and diagnostic criteria of hip-related pain in young and middle-aged active adults from the international hip-related pain research network, Zurich 2018. Br J Sports Med.

[10] Reiman M.P., Agricola R., Kemp J.L. (2021). Infographic. Consensus recommendations on the classification, definition and diagnostic criteria of hip-related pain in young and middle-aged active adults from the international hip-related pain research network, Zurich 2018. Br J Sports Med.

[11] Kemp J.L., Risberg M.A., Schache A.G., Makdissi M., Pritchard M.G., Crossley K.M. (2016). Patients with chondrolabral pathology have bilateral functional impairments 12 to 24 months after unilateral hip arthroscopy: a cross-sectional study. J Orthop Sports Phys Therapy.

[12] Kemp J.L., MacDonald D., Collins N.J., Hatton A.L., Crossley K.M. (2015). Hip arthroscopy in the setting of hip osteoarthritis: systematic review of outcomes and progression to hip arthroplasty. Clin Orthop Relat Res.

[13] Griffin D., Wall P., Realpe A. (2016). UK FASHIoN: feasibility study of a randomised controlled trial of arthroscopic surgery for hip impingement compared with best conservative care. Health Technol Assess (Rockv).

[14] Rhon D.I., Greenlee T.A., Marchant B.G., Sissel C.D., Cook C.E. (2019). Comorbidities in the first 2 years after arthroscopic hip surgery: substantial increases in mental health disorders, chronic pain, substance abuse and cardiometabolic conditions. Br J Sports Med.

[15] Hunter D.J., Eyles J., Murphy N.J. (2021). Multi-centre randomised controlled trial comparing arthroscopic hip surgery to physiotherapist-led care for femoroacetabular impingement (FAI) syndrome on hip cartilage metabolism: the Australian FASHIoN trial. BMC Musculoskelet Disord.

[16] Kemp J.L., Mosler A.B., Hart H. (2020). Improving function in people with hip-related pain: a systematic review and meta-analysis of physiotherapist-led interventions for hip-related pain. Br J Sports Med.

[17] Kemp J.L., Risberg M.A., Mosler A. (2020). Physiotherapist-led treatment for young to middle-aged active adults with hip-related pain: consensus recommendations from the International Hip-related Pain Research Network, Zurich 2018. Br J Sports Med.

[18] I R de Oliveira B., Smith A.J., O'Sullivan P.P.B. (2020). My hip is damaged’: a qualitative investigation of people seeking care for persistent hip pain. Br J Sports Med.

[19] Kemp J.L., Johnston R.T.R., Coburn S.L. (2021). Physiotherapist-led treatment for femoroacetabular impingement syndrome (the PhysioFIRST study): a protocol for a participant and assessor-blinded randomised controlled trial. BMJ Open.

[20] Tong A., Sainsbury P., Craig J. (2007). Consolidated criteria for reporting qualitative research (COREQ): a 32-item checklist for interviews and focus groups. Int J Q Health Care.

[21] Griffin D.R., Dickenson E.J., O'Donnell J. (2016). The Warwick agreement on femoroacetabular impingement syndrome (FAI syndrome): an international consensus statement. Br J Sports Med.

[22] Atkins L., Francis J., Islam R. (2017). A guide to using the theoretical domains framework of behaviour change to investigate implementation problems. Implement Sci.

[23] Scholes M.J., King M.G., Crossley K.M. (2021). The validity, reliability, and responsiveness of the international hip outcome tool–33 (iHOT-33) in patients with hip and groin pain treated without surgery. Am J Sports Med.

[24] Impellizzeri F.M., Jones D.M., Griffin D. (2020). Patient-reported outcome measures for hip-related pain: a review of the available evidence and a consensus statement from the International Hip-related Pain Research Network, Zurich 2018. Br J Sports Med.

[25] Barton C.J., Ezzat A.M., Bell E.C., Rathleff M.S., Kemp J.L., Crossley K.M. (2021). Knowledge, confidence and learning needs of physiotherapists treating persistent knee pain in Australia and Canada: a mixed-methods study. Physiother Theory Pract.

[26] Ezzat A.M., Bell E., Kemp J.L. (2022). “Much better than I thought it was going to be”: telehealth delivered group-based education and exercise was perceived as acceptable among people with knee osteoarthritis. Osteoarthritis Cartilage Open.

[27] Braun V., Clarke V. (2019). Reflecting on reflexive thematic analysis. Qual Res Sport, Exercise Health.

[28] Braun V., Clarke V. (2021). To saturate or not to saturate? Questioning data saturation as a useful concept for thematic analysis and sample-size rationales. Qual Res Sport, Exercise Health.

[29] Maxwell C., Robinson K., McCreesh K. (2021). Understanding shoulder pain: a qualitative evidence synthesis exploring the patient experience. Phys Ther.

[30] Snelgrove S., Liossi C. (2013). Living with chronic low back pain: a metasynthesis of qualitative research. Chronic Illn.

[31] Wallis J.A., Taylor N.F., Bunzli S., Shields N. (2019). Experience of living with knee osteoarthritis: a systematic review of qualitative studies. BMJ Open.

[32] Heerey J.J., Kemp J.L., Mosler A.B. (2018). What is the prevalence of imaging-defined intra-articular hip pathologies in people with and without pain? A systematic review and meta-analysis. Br J Sports Med.

[33] Mascarenhas V.V., Rego P., Dantas P. (2016). Imaging prevalence of femoroacetabular impingement in symptomatic patients, athletes, and asymptomatic individuals: a systematic review. Eur J Radiol.

[34] Heerey J., Agricola R., Smith A. (2020). The size and prevalence of bony hip morphology does not differ between football players with and without hip and/or groin pain: findings from the FORCe cohort. J Orthop Sports Phys Ther.

[35] Tak I., Glasgow P., Langhout R., Weir A., Kerkhoffs G., Agricola R. (2016). Hip range of motion is lower in professional soccer players with hip and groin symptoms or previous injuries, independent of cam deformities. Am J Sports Med.

[36] Mosler A.B., Weir A., Serner A. (2018). Musculoskeletal screening tests and bony hip morphology cannot identify male professional soccer players at risk of groin injuries: a 2-year prospective cohort study. Am J Sports Med.

[37] Timmins K.A., Leech R.D., Batt M.E., Edwards K.L. (2017). Running and knee osteoarthritis: a systematic review and meta-analysis. Am J Sports Med.

[38] Khan M.C.M., O'Donovan J., Charlton J.M., Roy J.S., Hunt M.A., Esculier J.F. (2021). The influence of running on lower limb cartilage: a systematic review and meta-analysis. Sports Med.

[39] da Costa B.R., Reichenbach S., Keller N. (2017). Effectiveness of non-steroidal anti-inflammatory drugs for the treatment of pain in knee and hip osteoarthritis: a network meta-analysis. The Lancet.

[40] Abbott J.H., Wilson R., Pinto D., Chapple C.M., Wright A.A. (2019). Incremental clinical effectiveness and cost effectiveness of providing supervised physiotherapy in addition to usual medical care in patients with osteoarthritis of the hip or knee: 2-year results of the MOA randomised controlled trial. Osteoarthritis Cartilage.

[41] Bull F.C., Al-Ansari S.S., Biddle S. (2020). World Health Organization 2020 guidelines on physical activity and sedentary behaviour. Br J Sports Med.

[42] Kunstler B.E., Cook J.L., Freene N. (2018). Physiotherapist-led physical activity interventions are efficacious at increasing physical activity levels: a systematic review and meta-analysis. Clin J Sport Med.

[43] Alentorn-Geli E., Samuelsson K., Musahl V., Green C.L., Bhandari M., Karlsson J. (2017). The association of recreational and competitive running with hip and knee osteoarthritis: a systematic review and meta-analysis. J Orthop Sports Phys Ther.

[44] Braaten A.D., Hanebuth C., McPherson H. (2021). Social determinants of health are associated with physical therapy use: a systematic review. Br J Sports Med.

