# Supplementary material for: What are participant beliefs regarding physical therapy led treatment? A qualitative study of people living with femoroacetabular impingement syndrome
Source: Braz J Phys Ther. 2024 May 21;28(3):101077. doi: 10.1016/j.bjpt.2024.101077 (PMC11215951; doi:10.1016/j.bjpt.2024.101077)
Supplement: Supplementary file 1 [file mmc1.pdf]

<u>Physio FIRST semi structured interview- topic guide</u>

Theme 1: Validity of the IHOT-33 questionnaire (note: the questionnaire should be sent to participant a few days before interview, and ask them to read it and consider whether there are any questions that (i) are not relevant to them; (ii) are important but were not included in the questionnaire that should have been included; and (iii) they don't understand)

1. Did you read the questionnaire that was sent to you? If not, take a minute to read it now
    a. Were there any questions that were not relevant to you in the questionnaire
    b. Were there any important things that you think were not included in the questionnaire that should have been included?
    c. Were there any questions that you did not understand?

Theme 2: Expectations

2. What is your understanding of what is leading to your hip pain?
3. What outcomes do you expect from having physiotherapy for your hip?
4. What do you think is a reasonable commitment to physiotherapy to gain?
    a. Visits- frequency
    b. Exercises- frequency, duration
    c. Cost
5. What barriers would you face to achieve that level of commitment?
6. What do you think would help you to achieve that level of commitment?
7. Which things would like to be educated about in regards to your hip pain?

Theme 3: Additional treatment options

1. Have you used any other methods of treatment for your hip pain?
    a. Medications
    b. Surgery
2. Have you seen any other health professionals for your hip pain?

Theme 4: Physical activity

8. What was your usual physical activity routine before COVID-19?
    d. Do you think
9. What does your physical activity involve now?
    e. How do you think COVID-19 has impacted your physical activity levels? (increased/decreased?)
10. Does your hip pain impact your level of physical activity- if yes, how?
11. How much physical activity and exercise do you think your hip needs?
    f. (if not raised, "Do you think you can do too much or too little exercise/physical activity for your hip? Tell me more about that")
